# Late consequences of chemotherapy on left ventricular function in women with breast cancer

**DOI:** 10.22088/cjim.13.3.511

**Published:** 2022

**Authors:** Maryam Nabati, Ghasem Janbabai, Mohammadreza Najjarpor, Jamshid Yazdani

**Affiliations:** 1 Department of Cardiology, Faculty of Medicine, Mazandaran University of Medical Sciences, Cardiovascular Research Center, Sari, Iran; 2 Department of Hematology, Gastrointestinal Cancer Research Center, Mazandaran University of Medical Sciences, Sari, Iran; 3 Student Research Committee, Faculty of Medicine, Mazandaran University of Medical Sciences, Sari, Iran; 4 Department of Biostatics, Faculty of Health, Mazandaran University of Medical Sciences, Sari, Iran

**Keywords:** Cancer, Cardiotoxicity, Chemotherapy, Left ventricular dysfunction, Strain

## Abstract

**Background:**

Cardiovascular disease is the main cause of death among breast cancer survivors. Several chemotherapy drugs may cause cardiovascular toxicity. Our study aimed to assess the late effects of chemotherapy on left ventricular (LV) systolic and diastolic function in a group of female breast cancer survivors.

**Methods:**

Our study was a case-control study consisted of 60 breast cancer survivors who had undergone chemotherapy for more than 5 years and a control group of 49 women without breast cancer. All patients underwent echocardiography and left ventricular ejection fraction (LVEF), global longitudinal strain (GLS), pulse-Doppler early transmitral peak flow velocity (E wave), early diastolic (e'), and left atrial (LA) diameter were calculated.

**Results:**

The mean LVEF and GLS were reduced in chemotherapy group (51.63±7.93% vs. 55.37±3.50%, P=0.002 and -17.99±3.27% vs. -19.25±2.27%, P=0.025). Also, the chemotherapy group had a larger left ventricular end-systolic internal diameter than the control group (1.74±0.44cm/m^2^ vs. 1.58±0.22cm/m^2^, P= 0.011). Logistic regression analysis showed among the different cardiovascular risk factors, chemotherapy had an association with decreasing LVEF.

**Conclusion:**

Breast cancer survivors might have an excess risk of having subclinical LV dysfunction over time. These findings present the potential benefits of echocardiographic assessment in breast cancer survivors.

Breast cancer is the most commonly diagnosed cancer and the second cause of death among women (1). Cardiovascular disease is the main cause of death among breast cancer survivors. Several chemotherapy drugs may cause cardiovascular toxicity (2). Chemotherapy-induced cardiotoxicity may develop several years to decades after the last chemotherapy dose (3). Antracyclines can cause dose-related and irreversible left ventricular (LV) dysfunction manifesting early or late after exposure. Also, cyclophosphamide is a commonly used alkylating agent in the treatment of breast cancer which can result in cytotoxicity and myocyte death. HER2-targeted therapies (human epidermal growth factor receptor 2) by trastuzumab (herceptin) can result in LV dysfunction and heart failure (HF) that is usually reversible. Antimicrotubule molecules, such as taxanes, may produce cardiac heart failure, rhythm and conduction disturbances, and ischemia. On the other hand, thoracic radiotherapy carries a significant risk of cardiotoxicity. The most common cardiac side effect of chemotherapy is LV systolic dysfunction (4, 5). A reduction in LV ejection fraction (LVEF) is usually representative of an extensive loss of function beyond capacity of the myocardium to compensate. Global longitudinal strain (GLS) is helpful in detecting subclinical alterations during chemotherapy, radiation and trastuzumab therapy (6).

There is a lack of controlled long-term studies regarding the incidence of undiagnosed cardiac dysfunction in adult female breast cancer survivors. Late cardiac dysfunction may be subclinical with a gradual onset and unknown prevalence with no established interventions to manage it. On the other hand, its early diagnosis is valuable because timely treatment can improve prognosis (7). The aim of our study was to assess the long-term effects of chemotherapy on LV function in a group of female breast cancer survivors by 2D conventional echocardiography as well as speckle-tracking echocardiography and comparing them with a control group. Our survivors had undergone chemotherapy for more than 5 years which is a longer time in comparison with previous studies.

## Method

Our study was a case-control study consisted of female breast cancer survivors and an age-matched control group of women without breast cancer. We reviewed the medical records of 67 female breast cancer survivors who had undergone chemotherapy for more than 5 years. After the exclusion of seven patients who had heart failure symptoms or LV dysfunction (an LVEF of less than 50%) early after chemotherapy, 60 patients were included in the study. Exclusion of these 7 patients was due to the fact that they may already have received some HF therapy with some confounding effects on echocardiographic variables. Also, the aim of our study was to evaluate the late consequences of chemotherapy. Therefore, these patients with known early cardiotoxicity were excluded from the study. The control group consisted of 49 age-matched women without breast cancer who were referred to our clinic for cardiovascular assessment. This study was performed according to the guidelines of the Helsinki Declaration and was approved by the ethics committee of our university. This research was performed in the oncology department of our university between 2019 and 2020. 

Written informed consent was obtained from all participants. The patients were divided into two groups: one group included a control group of 49 women without breast cancer and other groups consisted of 60 women with a prior history of breast cancer who were diagnosed to have cancer for more than 5 years prior to the date of screening and had undergone chemotherapy 7.22±3.49 years ago. Control group was selected among the women who had come for cardiovascular check-ups (referred for managing cardiovascular risk factors or exploring cardiovascular symptoms). An exercise test or myocardial perfusion SPECT was performed for all patients. If there was any evidence of ischemia, coronary angiography would be planned. Coronary atherosclerosis severity was determined by quantitative coronary angiography method. Patients with significant coronary stenosis requiring revascularization were excluded from the study. A coronary stenosis of more than 50% in the left main coronary artery or more than 70% in an epicardial artery was considered to be significant (8). Exclusion of patients with CAD was due to the fact that ischemia may have some confounding effects on LV systolic and diastolic function. Patients with recent chemotherapy due to cancer recurrence or LV dysfunction early after chemotherapy based on patients’ medical records have also been `excluded from the study. 

Patients' demographic and medical data, a list of all chemotherapy agents in the regimen and the number of cycles and individual cumulative dosage, the total number of radiotherapy sessions and receiving or not receiving trastuzumab treatment were recorded. Hypertension (HTN) was defined as a systolic blood pressure ≥ 140 mmHg, diastolic blood pressure ≥ 90 mmHg, or need to take antihypertensive medications (9). Diabetes mellitus (DM) was defined based on the guidelines of the American Diabetes Association or requiring to take oral hypoglycemic agent or insulin (10). Body mass index (BMI) was determined as a person's body weight in kilograms divided by height in meters squared. 

**Echocardiography: **All patients underwent transthoracic echocardiography by an ACUSON SC2000 with a 4V1c transducer (Siemens Medical Solutions USA Inc., Mountain View, CA). The images and movies were recorded during three consecutive beats and were stored on a hard disk for off-line analysis which were interpreted by a single expert echocardiographer blind to the patients’ data. The standard grayscale apical LV views were obtained as follows: 4-chamber, 2-chamber, and 3-chamber views. We measured the peak systolic longitudinal strain values in the basal, mid-, and apical segments of the septal, anterolateral, inferior, anterior, inferolateral, and anteroseptal walls. The endocardial and epicardial borders were tracked and the 2-chamber and 4-chamber left ventricular end diastolic volume (LVEDV), left ventricular end systolic volume (LVESV), and apical 2-chamber, 4-chamber, and 3-chamber global longitudinal strain (GLS) were calculated by a software dedicating for a semiautomated analysis (eSie VVI software). LVEDVs and LVESVs were indexed to body surface area. The peak GLS was calculated as the average from all 18 myocardial segments. The left ventricular ejection fraction (LVEF) was determined by subtracting LVESV from LVEDV and then dividing by LVEDV from the LV apical 4- and 2-chamber views by modified Simpson's technique. We measured end-systolic left ventricular internal diameter (LVIDs) and end-diastolic left ventricular internal diameter (LVIDd) perpendicular to the LV long axis, at the level of the mitral valve leaflet tips, in a parasternal long-axis view. The left atrial (LA) diameter was measured as the end-systolic vertical distance between the leading edge of the posterior aortic wall and the leading edge of the posterior LA wall in the parasternal long-axis view. The transmitral Doppler early diastolic velocity (E wave), the average of tissue Doppler mitral annulus septal and lateral early diastolic velocities (e'), and the E/e' ratio was calculated (11). The reproducibility of the LVEF and GLS calculations was determined by repeating the calculations in 10 patients within 24 hours; the intraobserver correlation coefficients were 0.92 and 0.94, respectively.

**Statistical analysis: **Continuous variables were described by the mean± standard deviation, and the categorical variables were represented as frequency and percentile. The Shapiro-Wilk test showed the variables were normally distributed. Continuous variables were compared by an independent t-test and categorical ones by chi-square and Fisher's exact tests. Also, a multiple linear regression analysis was used to modulate confounding effects of other variables on LVEF in whole individuals. A p- value less than 0.05 was considered statistically significant. SPSS/PASW (Predictive Analytics SoftWare) Statistics 18 (SPSS Inc., Chicago, IL) was used for analyzing the data. The minimum sample size was calculated based on the results presented by Ho et al. as follows (according to GLS value): 

## Results

In the group of breast cancer patients who underwent the scheduled chemotherapy, all patents had received cyclophosphamide, 56 (93.33%) patients had been treated with adriamycin, 10 (16.66%) patients had also received paclitaxel and 4 (6.66%) patients received Taxoter instead of adriamycin. Sixteen patients had received several courses of trastuzumab following chemotherapy (26.66%) and 50 (83.33%) patients had undergone adjutant radiotherapy. Anthropometric variables of the two study groups, the type and dose of chemotherapy agents and the number of radiotherapy treatment courses are presented in table 1. There was no statistically significant difference in the mean age, BMI, and the prevalence of HTN and DM between two groups (P=0.969, 0.064, 0.143, and 0.145, respectively). Echocardiographic variables of the two study groups are presented in table 2. 

**Table 1:** Anthropometric variables, the type and dose of chemotherapy agents and the number of radiotherapy treatment courses in the study population categorized by receiving or not receiving chemotherapy

	**Control group (n=49)**	**Chemotherapy group (n=60)**	**P-value**
Age (years)	54.13±9.53	54.20±9.27	0.969
HTN, n (%)	27 (54%)	24 (40%)	0.143
DM, n (%)	9 (18%)	18 (30%)	0.145
BMI (kg/m^2)^	29.94±5.04	28.52±2.55	0.064
Adriamycin dose/BSA (mg/m^2^)		443.32±95.66	
Cyclophosphamide/BSA dose (mg/m^2^)		4532.54±1113.79	
Taxoter (Docetaxel) dose/BSA (mg/m^2^)		337.94±299.66	
Paclitaxel dose/BSA (mg/m^2^)		405.57±113.20	
Trastuzumab, n (%)		16 (26.6%)	
Times of radiotherapy		23.89±5.99	
Last chemotherapy dose, months		86.11±39.42	

HTN: Hypertension; DM: Diabetes mellitus; BMI: Body mass index

**Table 2:** Echocardiographic variables in the study population categorized by receiving or not receiving chemotherapy

	**Control group (n=49)**	**Chemotherapy group (n=60)**	**P- value**
LVIDd/BSA (cm/m^2^)	2.74±0.21	2.77±0.34	0.618
LVIDs/BSA (cm/m^2^)	1.58±0.22	1.74±0.44	0.011
LVEF (%)	55.37±3.50	51.63±7.93	0.002
LVEF<55%, n (%)	1 (2%)	18 (30%)	<0.001
LA diameter (cm)	3.89±0.43	3.74±0.42	0.063
E wave velocity (m/s)	0.64±0.16	0.66±0.15	0.439
e' velocity (cm/s)	8.81±2.37	9.17±2.42	0.444
E/e'	7.63±1.81	7.55±1.99	0.828
GLS (%)	-19.25±2.27	-17.99±3.27	0.025
GLS>-16%, n (%)	2 (4.8%)	12 (20.3%)	0.026
Average apical four chamber LS (%) Average apical two chamber LS (%) Average apical three chamber LS (%)	-18.13±2.96 -19.57±3.59 -19.57±3.36	-17.92±4.25 -17.85±3.54 -18.54±4.51	0.893 0.023 0.228

LVIDd: End diastolic left ventricular internal diameter; LVIDs: End systolic left ventricular internal diameter; LVEF: Left ventricular ejection fraction; GLS: global longitudinal strain; LA: left atrium; E/e': Transmitral Doppler early diastolic velocity/mitral annular early diastolic velocity; LS: Longitudinal strain; BSA: Body surface area

**Figure 1:**
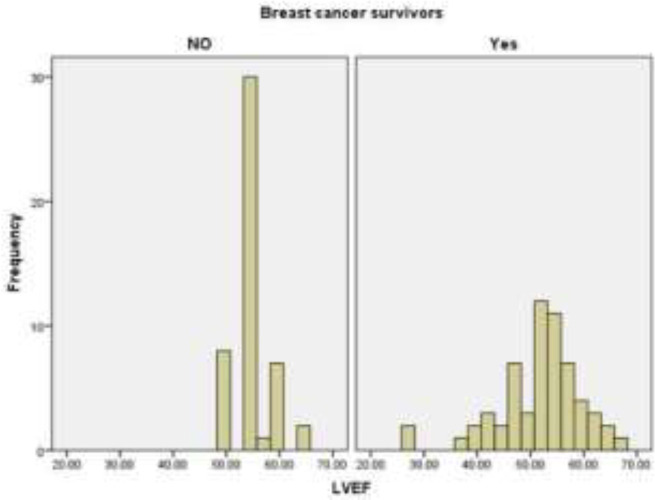
Histogram of the mean LVEF in breast cancer survivors and control group (P-value= 0.002)

**Figure 2:**
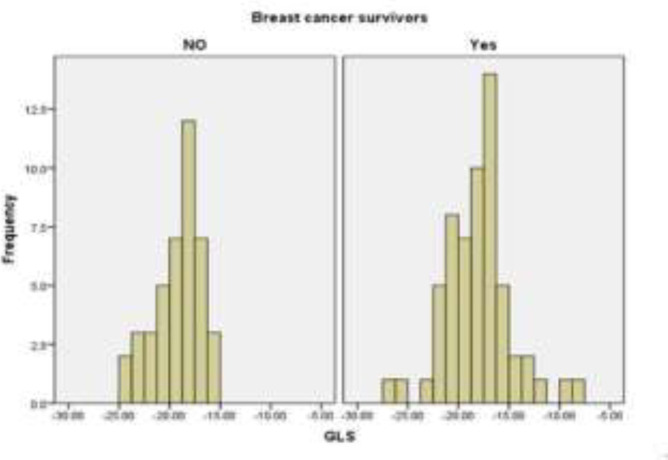
Histogram of the mean GLS in breast cancer survivors and control group (P=0.025)

The mean LVEF, GLS, and average apical two chamber longitudinal strain (LS) were significantly reduced in chemotherapy (51.63±7.93% vs. 55.37±3.50%, P = 0.002, -17.99±3.27% vs. -19.25±2.27%, P=0.025, and -17.85±3.54% vs. -19.57±3.59%, P= 0.023; figures 1, 2). 

The lowest value of LVEF was 27% in chemotherapy group and 50% in control group. One (2%) patient in control group and 18 (30%) patients in cancer survivors had an LVEF<50% (p-value<0.001). Two (4.8%) patients in control group and 12 (20.3%) patients in cancer survivors had a GLS>-16% (P =0.026). The lowest value of GLS was -8.30% in chemotherapy group and -15.94% in control group. Also, the chemotherapy group had a larger LVIDs/BSA than the control group (1.74±0.44cm/m^2^ vs. 1.58±0.22cm/m^2^, P= 0.011). LVIDd/BSA, E wave, e' velocity, E/e' ratio, average apical four and three chamber LS, and LA diameter were not significantly different between two groups.

Among the patients who received chemotherapy, LVEF was not different between trastuzumab treated and non-treated patients (-50.90±7.22% vs. -51.89±8.23%, P=0.672). We conducted a multiple linear regression analysis to modulate confounding effects of other variables on LVEF in whole individuals. The results showed among different risk factors, only chemotherapy was associated with decreasing LVEF in whole population. (Table 3).

**Table 3:** Independent association between cardiovascular risk factors and LVEF by multiple linear regression analysis

**Model**	**Unstandardized Coefficients**		**Standardized Coefficients**	**t**	**Sig.**	**95.0% Confidence Interval for B**	
	**B**	**Std. Error**	**Beta**			**Lower Bound**	**Upper Bound**
(Constant)	59.547	4.357		13.667	.000	50.904	68.190
Chemotherapy	-3.707	1.306	-.272	-2.838	.005	-6.298	-1.115
age	.086	.073	.118	1.165	.247	-.060	.231
HTN	-2.500	1.480	-.185	-1.689	.094	-5.436	.437
DM	-1.258	1.597	-.081	-.788	.433	-4.427	1.910

HLP: Hyperlipidemia; HTN: Hypertension; DM: Diabetes mellitus

## Discussion

 In our study,LVIDs/BSA was greater and LVEF and GLS were lower in chemotherapy group than control patients. Also, chemotherapy was a risk factor for decreasing LVEF. Some chemotherapy drugs induce cardiotoxicity by several mechanisms including rapid apoptosis or necrosis, growth inhibition, suppression of angiogenesis, or compromised repair capacity. Anthracyclines can cause cellular death by mitochondrial damage, inhibition of ATP production, cellular apoptosis, and production of free radical species. Trastuzumab can induce cardiotoxicity directly or by potentiating the cardiotoxic effect of anthracyclines. Cardiac adverse effect of trastuzumab is caused by decreasing ErbB2 receptor production. These receptors also present on myocardial cells and have some protective roles against myocardial injuries. On the other hand, taxanes can induce myocardial damage by direct effects on cellular organelles or releasing histamine which can lead to conduction disturbances and cardiac arrhythmias (5).Chemotherapy-induced cardiomyopathy can develop decades after treatment and has threefold higher mortality rate compared with idiopathic dilated cardiomyopathy (6). 

Cardiotoxicity is defined as a decrease in LVEF from >5% to <55%, accompanied with signs or symptoms of HF or an asymptomatic decrease in LVEF from >10% to <55% (11). Acute or subacute cardiotoxicity occurs during chemotherapy or up to 2 weeks after the completion of the therapy. Early onset chronic cardiotoxicity appears within 1 year after the completion of the chemotherapy and late onset chronic cardiotoxicity develops more than 1 year after the treatment. The most common manifestation of chronic cardiotoxicity is a subclinical, asymptomatic systolic or diastolic LV dysfunction that can cause irreversible heart failure and death. Functional and structural parameters of conventional echocardiography including LVEF and systolic and diastolic diameters and volumes are usually used for diagnosis of chemotherapy induced cardiotoxicity. LVEF is the most commonly used parameter for monitoring LV function. However, it may have low sensitivity for detecting small changes in LV function and cannot detect slight changes in myocardial contractility (12). However, LVEF is one of the most important parameters for detection and monitoring of cardiotoxicity in clinical practice which can help physicians make a decision about continuing or stopping chemotherapy (13). In our study, LVEF was lower in chemotherapy survivors than controls and a large number of cancer patients had an LVEF of less than 50%. Chronic doxorubicin-associated cardiomyopathy is related to the cumulative dose which is potentially irreversible, and may be life-threatening (14). 

The adverse cardiac effects of radiation may occur by many factors such as energy, fraction of dose, total dose, and the extent to which that organ is exposed to radiation. These harmful cardiac effects may occur early, or several years later (15). On the other hand, cyclophosphamide-induced cardiotoxicity is usually lethal. Its active metabolites can cause oxidative stress and direct endothelial capillary damage which is clinically manifested as acute heart failure and arrhythmia (16). Quantitative assessment of LV size by M-mode echocardiography is one of the most important predictors of subsequent HF and outcome (11). In our study, LVIDs was greater in chemotherapy group than control patients which may be a marker for increasing risk of future adverse outcome and developing HF. Non-Doppler two dimensional strain imaging by speckle tracking can efficiently evaluate myocardial deformation independent of angle, tethering and translational artefacts (5). The decrease in deformation indices occurs before deterioration in LVEF and may lead to earlier detection of subclinical myocardial dysfunction during long-term follow-up of cancer patients (3). In our study, GLS was -19.25% in control group and -17.99% in chemotherapy group. There was also a trend for the control to have a larger LA diameter than cancer patients in the present study. It may be explainable by the BMI trend and the fact that HTN was more prevalent in the control group than cancer survivors. 

 In 2014, Ho et al. evaluated the long-term effects of standard chemotherapy on myocardial function in 70 women who had received anthracycline treatment up to 6 years ago, and compared them with 50 healthy, normotensive women with no history of cardiovascular disease. They did not find any significant difference in LVEF between two groups. However, the chemotherapy group had reduced E/A ratios. Also, mitral annulus e' and s' velocities and GLS were significantly lower in chemotherapy group compared with healthy individuals (3).Our survivors had undergone chemotherapy for more than 5 years which is a longer time in comparison with aforementioned study. Therefore, chemotherapy patients may be at risk for decreasing LVEF and developing heart failure over time. Also, their control group was selected among healthy normotensive hospital staff members. Some of discrepancies between the results of the two studies may be explained by these issues. In our study, LS was significantly reduced in the apical two chamber but not in the apical four or three chambers. Small sample size may explain non-significant results of these variables. 

In 2017, Ganz et al. conducted a clinical trial on patients with breast cancer survivors who underwent anthracycline and taxane chemotherapy. The aim of this study was to compare the late cardiotoxicity of 297 patients who received trastuzumab with 110 patients without trastuzumab therapy at a median follow-up of 8.8 years. The results of their study showed the addition of trastuzumab to adjuvant anthracycline and taxane-based chemotherapy does not result in long-term worsening of cardiac function which was determined by measuring LVEF. These results were consistent with our study that LVEF was not different between trastuzumab treated and non-treated patients (17). Again in 2017, Boerman et al. performed a cross-sectional study on 350 women who survived breast cancer for at least 5 years after diagnosis and had undergone chemotherapy and/or radiotherapy and 350 aged-matched women. The primary outcome was an LVEF < 54% and an age-corrected LV diastolic dysfunction. Secondary outcomes included serum N-terminal pro B-type natriuretic peptide (NT-pro BNP) levels and newly diagnosed cardiovascular diseases. LVEF < 54% was present in 52 (15.3%) survivors and 24 (7%) controls (OR 2.4, 95%CI 1.4-4.0), but there was no significant increased prevalence of LV diastolic dysfunction. The level of serum NT-pro BNP increased and cardiovascular disorders were more prevalent among the survivors compared with control group (18). It is consistent with our study that a decrease in LVEF and GLS was more prevalent in cancer survivors than controls. However, the detection of GLS was not included in their study.

In 2020, Shahidsales conducted a prospective study on 31 female patients with left-sided breast cancer candidates for post-mastectomy radiotherapy following adriamycin-based chemotherapy regimen. The patients underwent echocardiography using speckle tracking and tissue Doppler imaging methods before the radiotherapy, six months, and one year after it. The results of their study showed that radiotherapy was associated with left heart diastolic dysfunction (manifested by E/e' ratio) and right heart systolic dysfunction especially in the first 6 months after radiotherapy. In comparison with our study, they evaluated net effects of radiotherapy on LV function in female patients with left sided breast cancer after one year. Their patients were compared with themselves. However, we evaluated late additive effects of both chemotherapy and radiotherapy in female breast cancer survivors. Also, our patients were compared with a control group (19). 

Again in 2020, Yu et al. performed a cross-sectional case-control study on 42 women with non-metastatic ERBB2-positive breast cancer who completed trastuzumab-based therapy, at least 2 years before study entry, with 38 of 42 patients (90%) were treated with anthracyclines before trastuzumab. These patients should meet 1 of the 2 criteria: cardiotoxicity (TOX group, n=22) developed during trastuzumab therapy manifested as asymptomatic decrease of LVEF>10% from baseline to <55%) or without any evidence of cardiotoxicity during trastuzumab treatment (NOTOX group, n = 20). Fifteen healthy control participants (HC group) were also entered into the study. Three groups were matched by the age. All patients underwent speckle-tracking echocardiography and maximal cardiopulmonary exercise testing. LVEF was significantly lower in the TOX (56.9%) group compared with the NOTOX (62.4%) and HC (65.3%) groups; GLS showed similar results (TOX group: −17.8%; NOTOX group: −19.8%; HC group: −21.3%) (p<0.001). Mean peak VO2 in the TOX group was 15% lower compared with the NOTOX group and 25%lower compared with the HC group (p<0.001) (20). Echocardiographic results were similar with the finding of our study. 

The limitation of present study were: small sample size and single center study. Also, our study was a case-control study comparing a group of breast cancer survivors with other group of control women instead of comparing themselves among themselves. Our research question was whether cancer exposure can cause or aggravate cardiovascular disorders. For future studies, three groups are suggested, including people with cardiovascular risk factors, cancer survivors, and healthy ones. A further limitation may be the impact of variation in specific drugs during treatments and current medications. Besides, we did not measure serum level of NT-pro BNP and BNP. Furthermore, the history of chest wall and or breast irradiation plus dose volume histogram of heart may affect the results, specifically in terms of cardiotoxicity in patients with left breast cancer.

 In Conclusion, Breast cancer survivors might have an excess risk of having subclinical LV dysfunction over time which warrants serial screening and close surveillance in this group of patients. These findings present the potential benefits of speckle-tracking echocardiography assessment in breast cancer survivors post chemotherapy treatment. 
